# Descriptive epidemiology of chronic liver disease in northeastern Italy: an analysis of multiple causes of death

**DOI:** 10.1186/1478-7954-11-20

**Published:** 2013-10-10

**Authors:** Ugo Fedeli, Elena Schievano, Manola Lisiero, Francesco Avossa, Giuseppe Mastrangelo, Mario Saugo

**Affiliations:** 1 SER – Epidemiological Department, Veneto Region, Passaggio Gaudenzio 1-35131 Padova (PD), Italy; 2 Department of Molecular Medicine, University of Padova, Via Giustiniani 2, 35128 Padova (PD), Italy

**Keywords:** Liver cirrhosis, Alcoholic liver disease, Hepatitis C, Mortality

## Abstract

**Background:**

The analysis of multiple causes of death data has been applied in the United States to examine the population burden of chronic liver disease (CLD) and to assess time trends of alcohol-related and hepatitis C virus (HCV)-related CLD mortality. The aim of this study was to assess the mortality for CLD by etiology in the Veneto Region (northeastern Italy).

**Methods:**

Using the 2008–2010 regional archive of mortality, all causes registered on death certificates were extracted and different descriptive epidemiological measures were computed for HCV-related, alcohol-related, and overall CLD-related mortality.

**Results:**

The crude mortality rate of all CLD was close to 40 per 100,000 residents. In middle ages (35 to 74 years) CLD was mentioned in about 10% and 6% of all deaths in males and females, respectively. Etiology was unspecified in about half of CLD deaths. In females and males, respectively, HCV was mentioned in 44% and 21% and alcohol in 11% and 26% of overall CLD deaths. A bimodal distribution with age was observed for HCV-related proportional mortality among females, reflecting the available seroprevalence data.

**Conclusions:**

Multiple causes of death analyses can provide useful insights into the burden of CLD mortality according to etiology among different population subgroups.

## Background

Mortality from liver cirrhosis has been declining in southern Europe over the last decades, possibly due to a reduction in alcoholic liver disease prevalence, a fall in the transmission of hepatitis C virus (HCV) infection, and vaccination campaigns against hepatitis B virus (HBV) [1,2]. Nonetheless, in a recent European report, the estimated HCV prevalence in the general Italian population was as high as 5.2% for the whole country, ranging from 2.4%-3.3% in northern regions to much higher values in central and southern Italy [3]. Hepatocellular carcinoma mortality is very high in Italy with respect to other European countries, with age standardized rates -world standard- equal to 6.72 and 1.92 per 100,000 in males and females, respectively [3]. Overall, about 60% of cirrhosis and hepatocellular carcinoma deaths were estimated to be attributable to HCV at the national level [3].

Usually mortality statistics are limited to the underlying cause of death, but a useful approach is represented by the analysis of all diseases reported on death certificates (multiple cause of death analysis), because it permits more extensive capture of chronic liver disease (CLD)-related deaths. When liver cirrhosis is mentioned on the death certificate, a related disease (e.g., liver cancer or viral hepatitis) or another disease category could be selected as the underlying cause. Moreover, such analysis allows the retrieval of the etiology of CLD. Although suffering from the underreporting of causes and types of liver diseases, the analysis of multiple causes of death data has been applied in US studies to examine time trends in HCV- and alcohol-related CLD mortality [4-7] and to assess the burden of CLD in high-risk subpopulations [8-10].

In the Veneto region (northeastern Italy) HCV seroprevalence is lower and alcohol consumption is higher than the national average [11,12], and this could influence both the overall burden and the etiology of CLD. The aim of this study was to examine the multiple causes of death database from the Veneto region in order to explore differences between analyses of the underlying cause and of multiple causes of death, to assess the most useful strategy in retrieving CLD-related deaths, and to describe the regional burden of CLD mortality according to etiology by gender, age class, and geographical area.

## Methods

The Veneto region has about 4,900,000 inhabitants and is subdivided into 21 local health units (LHUs) and 581 municipalities. Death certificates are sent by each municipality to the LHU, and the latter transmits a copy to the regional epidemiological department for the coding of causes of death according to the International Classification of Diseases, 10^th^ Edition (ICD-10). Since 2008, the electronic regional archive of mortality data is not limited to the underlying cause of death, but includes all diseases mentioned in the two parts of the death certificate. Part I includes conditions involved in the causal chain of events leading to death, from the underlying cause of death (e.g., liver cirrhosis) to the immediate cause of death (e.g., cardiac arrest). Part II includes other significant conditions that unfavorably influenced the course of the morbid process and thus contributed to the fatal outcome. Mortality statistics are based on internationally-adopted selection and modification rules that identify from all diseases reported on the death certificate a single underlying cause of death. The latter typically corresponds to the underlying cause stated in Part I; however, it could be also another disease reported in Part I or Part II or a derived condition [13]. In this study we used the following terminology: underlying cause of death for the disease selected according to international rules for mortality statistics and CLD-related mortality when the corresponding ICD-10 codes were retrieved from any part of the death certificate.

To identify CLD-related deaths, any ICD-10 code for conditions reported on the death certificate was selected according to three strategies:

•Strategy 1 included codes usually adopted in liver cirrhosis mortality statistics (K70, K73, K74).

•Strategy 2 added codes for chronic viral hepatitis, portal hypertension, and unspecified liver disease (B18, K76.6, and K76.9).

•Strategy 3 included all liver diseases and possible complications such as liver cancer, icterus, ascites, or esophageal varices (B15-B19, B94.2, I85.0, I98.2, C22, K70-K76, R17, R18), as in a recent report from Scotland [14].

Furthermore, ICD-10 codes were searched in the entire death certificate to classify the disease as relating to HBV (codes for acute or chronic infection: B16, B18.0, B18.1), HCV (codes for acute or chronic infection: B17.1, B18.2), or alcohol (codes for alcoholic liver diseases and for mental and behavioral disorders due to the use of alcohol: K70, F10).

To assess the most useful selection strategy, the total number of included deaths, the crude mortality rate, the proportion of deaths with identified etiology, and the distribution of the underlying cause of death were examined. To explore differences between common mortality statistics and multiple causes of death analysis, the number of deaths and crude rates were also computed by applying the three strategies of code selection only to the underlying cause.

Descriptive epidemiological measures were calculated for all CLD-related deaths and alcohol- and HCV-related mortality resulting from the best selection strategy. Crude and age-gender specific CLD-related mortality rates were computed as number of deaths per 100,000 population, with population data derived from the National Institute for Statistics (http://demo.istat.it/). Age-standardized rates were calculated by the direct standardization method according to different reference populations (World, European, and US 2000 standard). Lastly, in each gender and age class the proportional mortality (percentage of all registered deaths) and the share by etiology of total CLD-related deaths were computed.

To assess the geographical variability of HCV- and alcohol-related deaths, an indirect standardization method was applied to compute the expected number of deaths in each municipality based on regional rates. The observed to expected ratio (standardized mortality ratio, SMR) for each municipality was smoothed based on data from the nearest municipalities according to a Gaussian kernel function with a bandwidth equal to 10 kilometers [15], and data were mapped by quintiles of smoothed SMR.

## Results

Overall, 132,531 deaths were registered among residents in the Veneto region in the period from 2008 to 2010.

Table 1 shows the results of different selection strategies. If ICD-10 codes corresponding to strategy 1 were searched only in the underlying cause of death (as in usual liver cirrhosis mortality statistics), about 1.3% of overall deaths were selected, with a crude rate slightly above 10 per 100,000 residents. With the adoption of the multiple causes approach, even with the more restrictive strategy 1, at least 3.5% of total deaths could be related to CLD, with a crude mortality rate above 30 per 100,000 residents. The main etiological factors listed on death certificates were alcohol and HCV infection, with a small proportion of deaths attributable to HBV. About 1.6% of death certificates reported the coexistence of alcoholic liver disease and HCV (data not shown); about half did not specify any etiology. When including chronic viral hepatitis, portal hypertension, and unspecified liver disease, the mortality rate increased to about 40 per 100,000 and the share attributable to HCV reached 30%. If a broader spectrum of diseases possibly related to CLD was selected, the number of identified deaths doubled, and the share with unspecified etiology was close to 70%.

**Table 1:** Number of deaths and crude mortality rates obtained with different selection criteria (ICD-10 codes) by analysis of the underlying cause of death and of multiple causes of death; etiology of chronic liver diseases in the multiple causes of death analysis

	**Selection 1**	**Selection 2**	**Selection 3**
**ICD-10 codes selected**	**K70, K73, K74**	**B18, K70, K73, K74, K76.6, K76.9**	**B15-B19, B94.2, I85.0, I98.2, C22, K70-K76, R17, R18**
*Underlying cause of death analysis*			
Number of deaths	1694	2296	4141
Crude rate (× 10^5^)	11.6	15.7	28.3
*Multiple causes of death analysis*			
Number of deaths	4685	5941	9957
Crude rate (× 10^5^)	32.0	40.6	68.1
*Etiology, n (%):*			
Hepatitis C virus *(B17.1, B18.2)*	1000 (21%)	1756 (30%)	1763 (18%)
Hepatitis B virus *(B16, B18.0, B18.1)*	130 (3%)	258 (4%)	265 (3%)
Alcohol *(F10, K70)*	1215 (26%)	1221 (20%)	1233 (12%)
Other/not reported	2463 (53%)	2859 (48%)	6850 (69%)

Veneto region, 2008–2010.

**Figure 1:**
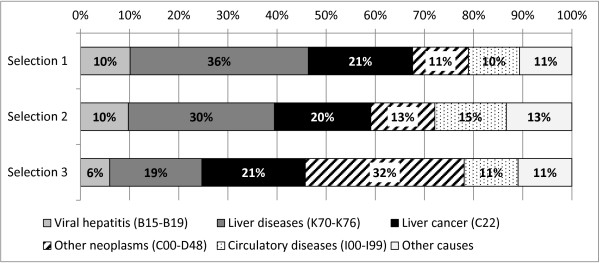
Multiple causes of death database, Veneto region, 2008–2010: underlying cause of death (ICD-10 codes) in chronic liver disease-related deaths retrieved by different identification strategies: Selection 1 (K70, K73, K74), Selection 2 (B18, K70, K73, K74, K76.6, K76.9), and Selection 3 (B15-B19, B94.2, I85.0, I98.2, C22, K70-K76, R17, R18).

Figure 1 shows that with selection strategies 1 and 2, viral hepatitis, liver cancer, and liver diseases were identified as the underlying cause of death in the majority of CLD-related deaths; it is worth noting that at least 20% of deaths had primary liver cancer identified as the underlying cause. If a broader range of ICD-10 codes was adopted (selection strategy 3), an underlying cause not related to liver conditions was identified in more than half of subjects; namely, deaths due to hepatic failure from liver metastases represented greater than 30% of included cases. The second strategy reported in Table 1 could therefore represent a compromise between sensitivity and specificity of the selection, allowing for the inclusion of all chronic viral hepatitis-related deaths but not of different systemic diseases leading to liver failure or dysfunction.

Table 2 shows different descriptive epidemiological measures for total CLD-related deaths and alcohol- and HCV-related mortality according to selection strategy 2. The few deaths with mention of both alcohol and HCV were analyzed both among HCV- and alcohol-related CLD. Overall, CLD represented almost 6% of all deaths in males and greater than 3% in females, but among those aged 35 to 74 years this share was as high as 10% and 6% in males and females, respectively. Age-standardized rates showed that the burden of all CLD and alcohol-related CLD was much higher in males, except for HCV-related mortality, which was only slightly higher than in females. Death rates reached high values in the 45 to 54 year age class, but thereafter the increase with age was much steeper among females, with a male to female ratio falling from 4:1 to 1.6:1 among elderly subjects. This different pattern was mainly due to HCV-related diseases: death rates were high in both genders among subjects aged 45 to 54 years with a male to female ratio of 5:1, and further increased in subjects 65 years or older, especially among females, with a male to female ratio falling below unity. In people aged 45 to 64 years, alcohol-related deaths represented a substantial proportion of all deaths (4% in males and 2% in females) and of CLD deaths (about one-third in both genders); in older age classes, they still accounted for a substantial burden of mortality only in males, but not in females.

**Table 2:** Age-specific number of deaths, mortality rates, proportion over all deaths and chronic-liver disease (CLD) deaths, crude and age-standardized rates: all CLD, hepatitis C virus (HCV)-related, and alcohol-related deaths, males and females of the Veneto region, 2008–2010

	**All CLD**		**HCV-related**		**Alcohol-related**	
	**Males**	**Females**	**Males**	**Females**	**Males**	**Females**
Crude rate (× 10^5^)	51.3	30.3	10.7	13.3	13.5	3.4
US 2000 standard (× 10^5^)	40.5	17.1	8.8	7.1	10.4	2.3
European standard (× 10^5^)	37.1	14.3	7.6	5.4	10.2	2.3
World standard (× 10^5^)	24.8	9.0	5.0	3.2	7.0	1.6
*Number of deaths*						
<35 yrs	13	9	7	4	-	-
35-44 yrs	107	36	47	18	34	10
45-54 yrs	348	89	106	21	104	34
55-64 yrs	702	193	88	25	249	63
65-74 yrs	1161	434	181	148	328	74
≥75 yrs	1345	1504	336	775	252	73
all ages	3676	2265	765	991	967	254
*Age-specific rates (*× *10*^*5*^*)*						
<35 yrs	0.5	0.3	0.3	0.2	-	-
35-44 yrs	8.3	2.9	3.6	1.5	2.6	0.8
45-54 yrs	32.5	8.5	9.9	2.0	9.7	3.2
55-64 yrs	80.5	21.5	10.1	2.8	28.6	7.0
65-74 yrs	165.5	54.2	25.8	18.5	46.8	9.2
≥75 yrs	274.4	169.4	68.6	87.3	51.4	8.2
*% all deaths*						
<35 yrs	1.1%	1.6%	0.6%	0.7%	-	-
35-44 yrs	9.3%	5.4%	4.1%	2.7%	2.9%	1.5%
45-54 yrs	13.4%	5.9%	4.1%	1.4%	4.0%	2.2%
55-64 yrs	11.3%	6.0%	1.4%	0.8%	4.0%	2.0%
65-74 yrs	8.9%	6.1%	1.4%	2.1%	2.5%	1.0%
≥75 yrs	3.4%	2.7%	0.8%	1.4%	0.6%	0.1%
all ages	5.8%	3.3%	1.2%	1.4%	1.5%	0.4%
*% CLD deaths*						
<35 yrs			54%	44%	-	-
35-44 yrs			44%	50%	32%	28%
45-54 yrs			30%	24%	30%	38%
55-64 yrs			13%	13%	35%	33%
65-74 yrs			16%	34%	28%	17%
≥75 yrs			25%	52%	19%	5%
all ages			21%	44%	26%	11%

**Figure 2:**
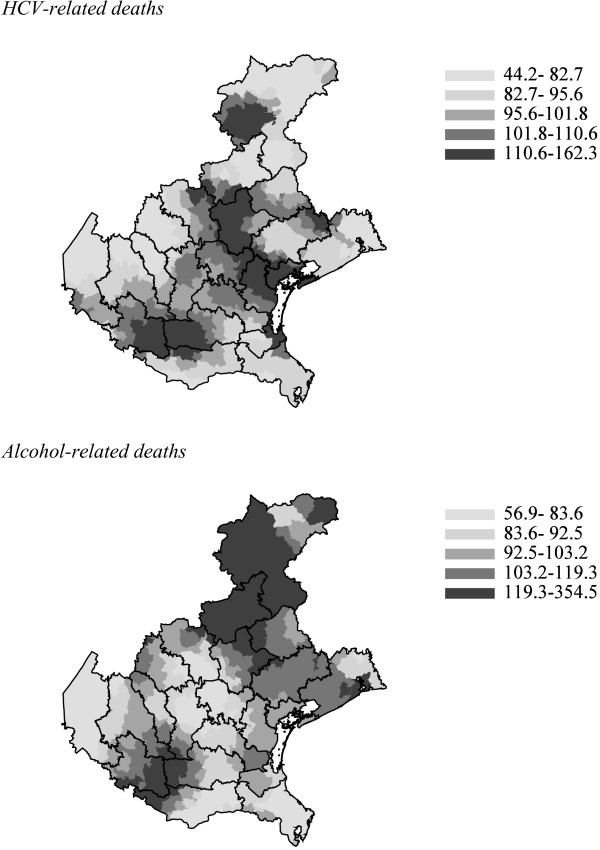
**Municipal data on hepatitis C virus (HCV)-related and alcohol-related chronic liver disease deaths, males of the Veneto region, 2008–2010.** Quintiles of smoothed standardized mortality ratios; 100 = observed deaths equal to those expected according to regional rates.

Figure 2 illustrates that among males the pattern of CLD mortality at the municipal level was different according to the etiology: there was an excess of alcohol-related mortality in the northeastern part of the region, whereas HCV-related mortality peaked in the central-coastal area. Among females, HCV-related mortality displayed a pattern similar to that in males (data not shown), whereas due to limited numbers no geographical analysis of alcohol-related deaths was carried out.

## Discussion

Analyses of multiple causes of death data demonstrated that CLD led to a great burden of mortality in the Veneto region, causing or contributing to about 10% of deaths among males under 75. The contribution of alcohol and HCV to overall CLD mortality was similar in men (about 20-25%) but not in women, where the proportion of HCV-related deaths (44%) was four-fold higher than that of alcohol-related CLD deaths (11%). Our finding that about half of total CLD deaths were without specification of etiology is similar to a figure reported in US data [10].

The multiple causes of death approach allowed a much greater burden of mortality to be attributed to CLD than usual statistics of liver cirrhosis deaths based only on the underlying cause. This is due to the inclusion of both a great number of deaths with primary liver cancer as the underlying cause and of deaths with an underlying cause listed in other disease categories. For the latter group, a substantial role of CLD in the fatal outcome could be questionable. However, it must be remarked that due to aging of the population in the Veneto region, about one-fourth of deaths in males and half in females occur in very elderly people over 84; in these subjects, it is often difficult to identify a single real cause of death on clinical grounds, and the multiple causes analysis could uncover the role of multiple comorbidities in the fatal process.

Selection strategy 2 was chosen to examine the patterns of CLD-related deaths by gender, age class, and geographical area; however, the selection of codes could be opportunely modulated based on the characteristics of the database at hand. It is worth noting that with the adopted strategy, hepatocellular carcinoma deaths without mention of CLD were not included, and this could lead to the problem of CLD-related mortality underestimation even with the multiple causes of death approach.

Alcohol-related liver diseases could be particularly affected by underreporting on death certificates because of the stigma associated with alcohol abuse. However, alcohol-related mortality rates found in the present study are similar to the figures reported among non-Hispanics in data from the US [10]. Furthermore, according to a survey carried out in male Veneto workers in the early 2000s [12], alcohol consumption was high in the areas with high alcohol-related CLD mortality shown in Figure 2. The adopted list of alcohol-related causes was somewhat restrictive; however, by examining more ICD-10 codes of neurological, cardiac, and other alcohol-related diseases, very few additional CLD deaths could be related to alcohol (data not shown).

In previous studies death certificates were linked to medical or laboratory records, demonstrating that HCV was mentioned in about two-thirds of death certificates of subjects with HCV infection in studies from the US [16,17] and in about half in one report from Scotland [14]. No validation of mortality data was carried out in the present study. However, HCV-related mortality rates were at least double of those reported in studies from the US [4,7,10]; especially among females mortality rates were more elevated than those registered in high-risk US sub-populations such as Hispanics [10]. On the other hand, the pattern of HCV-related proportional mortality, at least in females, reflected the findings of a seroepidemiological study recently carried out in Veneto, in which the distribution of anti-HCV antibodies was bimodal, with peaks at 35–44 years (2.1%) and ≥75 years (12%) [11]. The high peak in the elderly reflects a cohort effect of the epidemic of HCV infection in Italy in the 1950s due to the widespread use of nondisposable medical instruments. The smaller peak in younger subjects may be related to intravenous drug abuse, tattooing, and piercing [11]. Such a distribution shift towards the elderly cohorts could lead to a decline in both HCV prevalence and HCV-related mortality in the general population over the next decades. However, these figures individuate also a relevant issue in health care since clinicians are facing an increasing aging population with HCV-related CLD [11]. Moreover, the second smaller peak in the young and middle-aged classes needs to be strictly monitored.

Although HBV-related mortality was of limited magnitude, ongoing immigration from high prevalence countries could lead to an increasing burden of disease and deaths in the near future.

It is worth noting that in spite of the underreporting of etiology, such patterns are consistent with the results of prevalence studies in areas close to the Veneto region: in a community in northern Italy where the prevalence of CLD was ascertained by multiple data sources, HCV was the main cause in both genders, closely followed by alcohol abuse among males; about one out of four cases had indefinite etiology [18].

Death certificates lack information on other etiological factors such as nonalcoholic fatty liver disease (NAFLD) and genetic, occupational, and environmental risk factors [10]. NAFLD represents a growing phenomenon, and it is likely that it makes a significant contribution to CLD mortality [1]. NAFLD is known to be particularly prevalent in patients with diabetes [2], and in our records diabetes was reported in about 20% of CLD deaths without mention of etiology.

To our knowledge, the present study is the first to examine multiple causes of death for CLD outside the US and UK. The present analyses depict different patterns of alcohol-related and HCV-related CLD mortality by gender, age, and geographical area. Although confined to males among older age classes, alcohol-related mortality emerges as a substantial issue in middle-aged subjects of both genders, whereas HCV-related mortality constitutes an important concern in younger subjects and among the elderly.

## Conclusions

The multiple causes of death approach allowed to attribute to CLD a much greater burden of mortality than usual statistics of liver cirrhosis deaths based only on the underlying cause, and could be potentially more useful for the surveillance of CLD in the next years. To reduce underreporting and improve epidemiological information on the etiology of CLD, certifying physicians should be encouraged to fully compile death certificates. Bearing in mind their inherent limits, such analyses should be adopted whenever electronic archives with all the underlying and contributing causes of death are available.

## Abbreviations

HCV: Hepatitis C virus; HBV: Hepatitis B virus; CLD: Chronic liver disease; LHU: Local health units; ICD-10: International classification of diseases, 10^th^ edition; SMR: Standardized mortality ratio; NAFLD: Nonalcoholic fatty liver disease.

## Competing interests

The authors declare they have no competing interests.

## Authors’ contribution

UF, GM, and MS designed the study. FA, UF, ML, and ES collected data and performed statistical analyses. UF, ES, and FA wrote the first draft of the manuscript. ML, GM, and MS revised the paper. All authors read and approved the final manuscript.
